# Large‐Area Carbon Nanosheets Doped with Phosphorus: A High‐Performance Anode Material for Sodium‐Ion Batteries

**DOI:** 10.1002/advs.201600243

**Published:** 2016-09-12

**Authors:** Hongshuai Hou, Lidong Shao, Yan Zhang, Guoqiang Zou, Jun Chen, Xiaobo Ji

**Affiliations:** ^1^ State Key Laboratory for Powder MetallurgyCentral South UniversityChangsha410083China; ^2^ College of Chemistry and Chemical EngineeringCentral South UniversityChangsha410083China; ^3^ Shanghai Key Laboratory of Materials Protection and Advanced Materials in Electric PowerShanghai University of Electric PowerShanghai200090China

**Keywords:** anodes, carbon nanosheets, phosphorus‐doped, sodium‐ion batteries

## Abstract

Large‐area phosphorus‐doped carbon nanosheets (P‐CNSs) are first obtained from carbon dots (CDs) through self‐assembly driving from thermal treatment with Na catalysis. This is the first time to realize the conversion from 0D CDs to 2D nanosheets doped with phosphorus. The sodium storage behavior of phosphorus‐doped carbon material is also investigated for the first time. As anode material for sodium‐ion batteries (SIBs), P‐CNSs exhibit superb performances for electrochemical storage of sodium. When cycled at 0.1 A g^−1^, the P‐CNSs electrode delivers a high reversible capacity of 328 mAh g^−1^, even at a high current density of 20 A g^−1^, a considerable capacity of 108 mAh g^−1^ can still be maintained. Besides, this material also shows excellent cycling stability, at a current density of 5 A g^−1^, the reversible capacity can still reach 149 mAh g^−1^ after 5000 cycles. This work will provide significant value for the development of both carbon materials and SIBs anode materials.

## 1 Introduction

The large‐scale application of lithium‐ion battery (LIB) which is one of the most common rechargeable batteries may be gradually hampered by the insufficient lithium resource in earth cluster. Since the physicochemical property of sodium with abundant and low‐cost source is similar to that of lithium, sodium‐ion battery (SIB) has been considered as a promising rechargeable battery which can supplement or replace LIB in some fields.^1^ In view of the successful application of carbon material in LIBs, carbon material has been supposed to be a promising electrode candidate for SIBs.^2^ Because Na^+^ ions cannot be easily intercalated into the layers of graphite due to larger radius of Na^+^, resulting in a low capacity of 31 mAh g^−1^, conventional graphite might not be suitable for SIBs.^3^ However, various amorphous carbon materials including hollow nanostructured carbons,^4^ carbon nanospheres,^5^ carbon nanofibres,^6^ carbon nanosheets,^7^ porous carbons,^8^ hard carbon,^9^ graphene,^10^ and heteroatom‐doped carbon materials^11^ are being investigated as anode for SIBs in full swing, benefiting from disordered nanodomains with randomly oriented graphene layers and voids between these domains, which can provide rich active sites for storing Na^+^.^9^, ^12^ Unfortunately, lots of amorphous carbon‐based anode materials are accompanied with low initial columbic efficiency and poor specific capacity at high current density. For the practical use of these materials in commercial SIBs, the above‐mentioned issues must be addressed.

As reported,[[qv: 4b]] when the layer distances of graphite increase to 0.37 nm, the energy barrier for Na^+^ ion insertion would be low enough to conquer. Thus, to develop carbon materials with an interlayer distance over 0.37 nm is an effective way to facilitate the Na^+^ insertion. Introducing heteroatoms into carbon material is an effective approach to enlarge its interlayer distance, which is helpful to the Na^+^ insertion.^11^ Besides, heteroatoms doping has been also regarded as one of the most effective strategies to promote the electrochemical behaviors of carbon materials, owing to the tailoring of electronic properties and effects on electronic charge distribution of adjacent carbon atoms.^13^ In addition, 2D nanosheets often possess large exposed surfaces and specific facets, which can offer high active surface area for ions insertion, continuous conducting pathways for electrons, open shortened diffusion distance for ions and facile strain relaxation during battery operation, making them be ideal electrode materials for SIBs with high power and energy density.^14^ Nonetheless, producing high quality and large quantity of 2D nanomaterials, especially doped with heteroatoms, in a commercially viable way is still full of high challenges.

Quantum‐sized carbon dot is considered as a promising alternative to semiconductor quantum dots in various applications on account of its unique physicochemical characteristics.^15^ Nevertheless, the low‐yield, complicated, and expensive synthesis route largely hinder the practical application. In this work, we proposed a new approach to produce carbon dots (CDs) directly from acetaldehyde solution and NaOH in large scale within short time, further improving the operability and availability of CDs. Under the guarantee of high‐quality and high‐yield CDs precursor, the 2D carbon nanosheets doped with phosphorus (P‐CNSs) were constructed by calcining the mixture of CDs and NaH_2_PO_4_ under Ar atmosphere. Moreover, the produced P‐CNSs were successfully applied as anode material for SIBs, exhibiting remarkable electrochemical performances with excellent cycle stability and rate capability.

## 2 Results and Discussion

**Figure 1:**
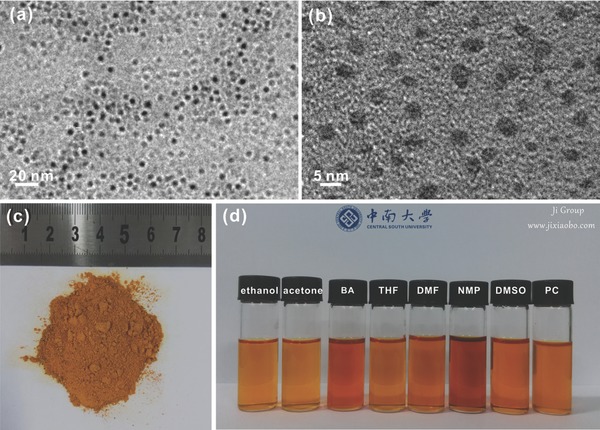
TEM images of CDs a,b) and optical photographs of CDs powder c) and solution in different solvents d).

**Figure 2:**
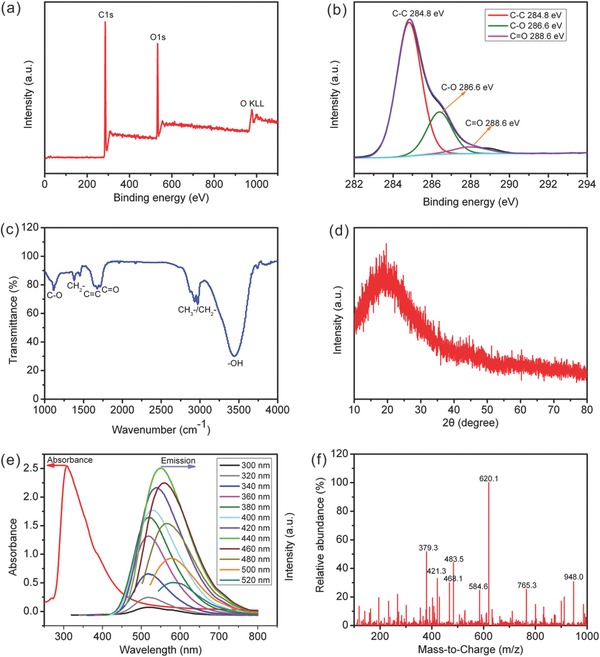
The survey XPS spectrum a), C1s high‐resolution XPS spectra b), FTIR spectrum c), XRD pattern d), UV absorption and excitation‐dependent PL spectra e), and MS spectrum f) of CDs.

**Figure** **1**a,b shows the transmission electron microscopy (TEM) images of the as‐prepared monodisperse CDs with diameters of 3.0–5.0 nm. Figure 1c,d displays the photos of brown CDs powders and solutions. Interestingly, CDs can be well dispersed in organic solvents, including ethanol, acetone, benzyl alcohol (BA), tetrahydrofuran, dimethyl formamide, 1‐methyl‐2‐pyrrolidinone, dimethyl sulfoxide, and propylene carbonate (PC). Moreover, these solutions are uniform, transparent, and stable, which may facilitate the application of CDs in more fields. X‐ray photoelectron spectroscopy (XPS, **Figure** **2**a,b) and Fourier transform infrared spectrum (FTIR, Figure 2c) reveal that the CDs contain rich oxygenous functional groups, such as C=O and C—OH, which might be in favor of its surface functionalization.^16^ The X‐ray diffraction (XRD) pattern (Figure 2d) with a broad peak centered at about 20° suggests the highly amorphous structure of obtained CDs. The absorption peaks at ≈310 nm in the UV–vis absorption spectrum (Figure 2e) is related with the n–π* electronic transition occurred on the surface of CDs, resulting from the C=O bond.^17^ The fluorescence emission spectra (Figure 2e) of CDs collected at various excitation wavelengths indicate that the CDs have a typical excitation‐dependent photoluminescence (PL) behavior, in good accordance with the reported CDs.^16^, ^17^, ^18^

Note that the aldol reaction would occur when aldehydes or ketones with α–H are placed under alkaline conditions. The acetaldehyde can be converted into unsaturated aldehydes, and then numerous oligomers are formed coming from the polymerization reaction of these unsaturated aldehydes. With such strong alkaline condition, some side reactions, including dehydration and dehydrogenation, would be conducted, leading to the formation of extended carbon chains, which may further curl and intertwine, resulting in the formation of carbon dots. The XPS and FTIR spectra (Figure 2a–c) have confirmed that the CDs are comprised of carbon and rich oxygen‐containing functional group, and the mass spectrometry result (Figure 2f) further shows that the CDs are composed of oligomers with different molecular weights.

**Figure 3:**
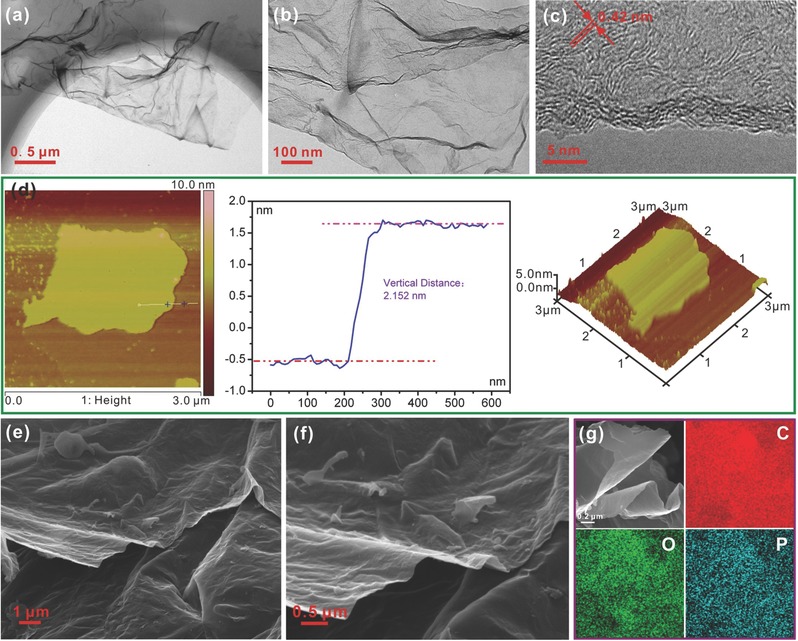
TEM a–c), AFM d), SEM e,f), and images and elemetal mappings g) of P‐CNSs.

The resultant CDs can be utilized to design and construct 2D phosphorus‐doped carbon materials through thermal treatment assisted by NaH_2_PO_4_. **Figure** **3**a,b shows the TEM images of the attained sample, it can be seen that the attained sample is consisted of ultrathin sheets with large area, which is quite similar to the reported graphene. Figure 3c presents the high‐resolution TEM (HRTEM) image, the expanded carbon lattice with an average distance of ≈0.42 nm can be clearly observed, and the enlarged interlayer distance might be more beneficial to the insertion of Na^+^ ions.[[qv: 4a]],[[qv: 11b]] And the atomic force microscope (AFM) is employed to measure the thickness of this sheet, the results are displayed in Figure 3d, indicating that the thickness is 2.152 nm. Scanning electronic microscopy (SEM) images (Figure 3e,f) further show the visual morphology of P‐CNSs under a wide range, the well‐defined ultrathin films with a size of several micrometres is displayed. Figure 3g depicts the elemental mappings of the P‐CNSs, suggesting that C, O, and P are uniformly distributed in the sample.

**Figure 4:**
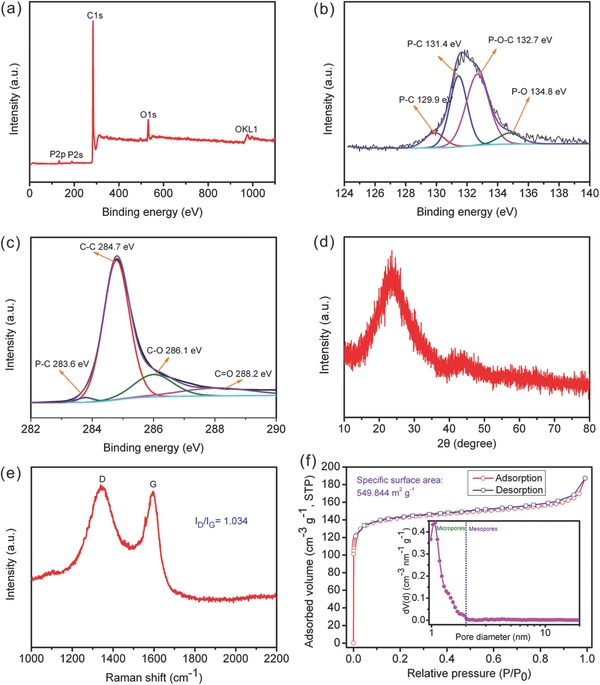
XPS spectra a–c), XRD pattern d), Raman spectrum e), and N_2_ adsorption–desorption isotherms and pore size distribution f) of P‐CNSs.

XPS is utilized to analyze the detailed chemical composition and the bonding configuration of phosphorus atoms in the P‐CNSs. The XPS results show that the P‐CNSs are mainly composed of C (90.92%), O (7.69%), and P (1.39%) (**Figure** **4**a and **Table** **1**). In the C1s spectrum (Figure 4b), the peaks at binding energies of 284.7, 286.1, and 288.2 eV are ascribed to C—C, C—O, and C=O, respectively.^17^ The peak located at 283.6 eV should be assigned to P—C, indicating that P atoms are indeed inserted into the carbon lattice.^19^ The high‐resolution P2p spectrum (Figure 4c) of P‐CNSs is also collected to get more insight into phosphorus doping; the peaks centered at 129.9 and 131.4 eV can be related to the P–C bonding,^20^ which powerfully demonstrates that P atoms are successfully incorporated into the carbon structure during the thermal annealing process. The peaks at 132.7 and 134.8 eV can be attributed to P—O—C and P—O (phosphate).^13^, ^20^ Two broad peaks at ≈23° and ≈44° appear in the XRD pattern (Figure 4d), corresponding to the (002) and (100) diffraction of disordered carbonaceous structure.[[qv: 4a]],[[qv: 11a]] The further structural analysis of P‐CNSs is conducted through Raman spectroscopy (Figure 4e), two distinct peaks centered at ≈1345 and ≈1594 cm^−1^ are considered as the D band (a series of structural defects) and G band (graphitic structures).[[qv: 10b]],[[qv: 11b]] The nitrogen adsorption–desorption technology (Figure 4f) is carried out to investigate the specific Brunauer–Emmett–Teller (BET) surface area and pore size of P‐CNSs, revealing a high specific surface area (SSA) of 549.844 m^2^ g^−1^ and small pore diameter of ≈1.2 nm for P‐CNSs, which can bring better electrolyte wetability and provide more active sites for sodium storage, thus facilitating Na^+^ diffusion.

**Table 1:** Elemental compositions and specific surface areas of samples carbonized at different temperatures and constant mass ratio of CDs to NaH_2_PO_4_ (1:10)

Samples	*C*_xps_ [At%]	*O*_xps_ [At%]	*P*_xps_ [At%]	*S*_BET_ [m^2^ g^−1^]
C‐400	89.20	10.28	0.52	21.996
C‐600	93.11	6.33	0.56	59.171
C‐800	93.28	5.97	0.75	189.609
C‐900 (P‐CNSs)	90.92	7.69	1.39	549.844

**Figure 5:**
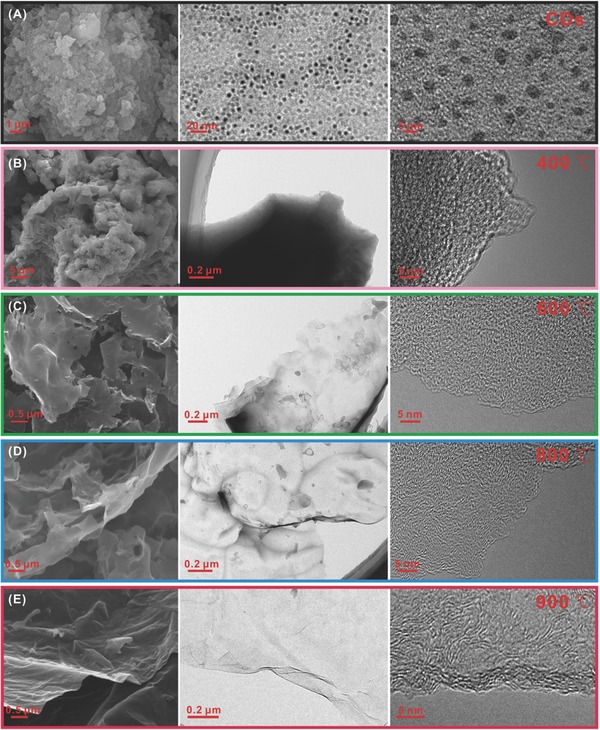
SEM and TEM images of CDs and the samples carbonized at different temperatures.

To gain insightful cognition of the chemical science hidden in the process from 0D CDs to 2D nanosheets, a series of conditional experiments on calcining temperature and mass ratio of CDs to NaH_2_PO_4_ are carried out. It was found that the temperature of thermal treatment is a key to determine the morphology and structure of carbon materials. Unexceptionally, in the CDs system, from the SEM and TEM images (**Figure** **5**), the change is observed with the increase of carbonizing temperature. At lower temperature of 400 °C, carbon chunk with highly amorphous texture is obtained (Figure 5B). At 600 °C, although the morphology is not very uniform, the rudiment of 2D carbon has been formed. Notably, from 400 to 600 °C, the highly amorphous structure does not change much (Figure 5C). When the calcining temperature reaches 800 °C, a thin sheet with large area can be clearly seen, and a few graphitized areas begin to appear (Figure 5D). When the temperature is further increased to 900 °C, an ultrathin sheet with larger area can be prepared with a thickness of 2 nm and some obvious lattice fingers are presented on the HRTEM image (Figure 5E). XRD patterns (Figure S1, Supporting Information) further demonstrate the change of crystal texture from 400 to 900 °C. In addition, the calcining temperature is also significant for the phosphorus content in as‐prepared P‐doped carbon nanosheets. XPS analyses (Figure S2, Supporting Information, and Table 1) show that P content is gradually increased from 0.52% to 1.39% with increasing temperature from 400 to 900 °C, which may be that high temperature is more beneficial to the decomposition of phosphate and the formation of P—C and P—O—C bonds. The CH_3_−/CH_2_− was detected in the C‐400 apart from –OH, C—O, and C=O, originating from the lack of sufficient energy for carbonization. Upon 600 °C, no obvious difference of functional groups is found (Figure S3, Supporting Information). The N_2_ absorption–desorption measurements (Figure S4, Supporting Information, and Table 1) reveal an increase in SSA with increasing temperature from 400 to 800 °C, especially between 800 and 900 °C.

**Figure 6:**
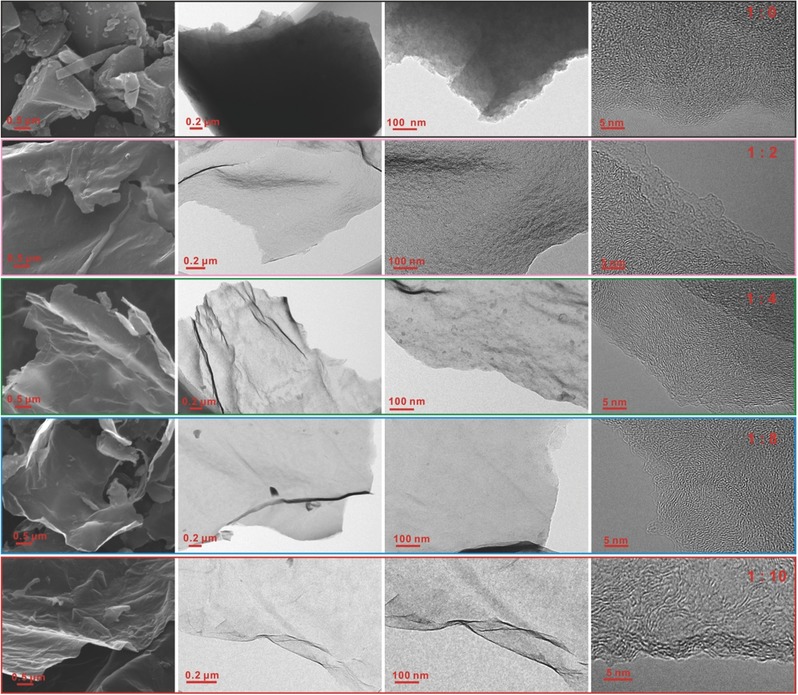
SEM and TEM images of samples carbonized at different mass ratios of CDs to NaH_2_PO_4_.

It has been demonstrated that calcining temperature was crucial for regulating the assembly and transformation processes during the formation of nanosheets. Besides, it can be noticed as well that the mass ratio of CDs to NaH_2_PO_4_ has an influence on the morphology, structure and composition of the calcined products. Details about the evolution process are also investigated. **Figure** **6** presents SEM and TEM images of the calcined products with various mass ratios from 1:0 to 1:10. Note that the added NaH_2_PO_4_ was essential for the formation of the nanosheets, controlling, and morphological evolution. Without NaH_2_PO_4_, the self‐assembling process to nanosheets was unavailable, only carbon chunk was produced. With increasing the ratio to 1:2, the sheet‐like morphology was observed, and the thickness of sheets is decreased with the increase of ratio, which is further confirmed by AFM (Figure S5, Supporting Information). HRTEM images (Figure 6) show that some graphitized domains randomly distributed in disordered carbon appear in all the samples, which are also confirmed by XRD (Figure S6, Supporting Information). According to XPS analysis (Figure S7, Supporting Information, and **Table** **2**), we can conclude that P content is gradually increased from 0.72% to 1.39% with increasing ratio from 1:2 to 1:10. The Raman spectra (Figure S8, Supporting Information) reveal that the D band gradually enhances with the increase of ratio, as a result of the introduced defects, further demonstrating the successful P doping. Since the content of doped P is very low, no obvious difference in the functional groups is noticed under different calcining temperatures from FTIR spectra (Figure S9, Supporting Information). The ratio has a significant influence on the BET SSA as well (Figure S10, Supporting Information, and Table 2). From 1:8 to 1:10, the SSA largely rises, which may be attributed to the decreased thickness (Figure S5, Supporting Information) and pore size.

**Table 2:** Elemental compositions and specific surface areas of samples carbonized at different mass ratios of CDs to NaH_2_PO_4_ and constant calcining temperature (900 °C)

Samples	*C*_xps_ [At%]	*O*_xps_ [At%]	*P*_xps_ [At%]	*S*_BET_ [m^2^ g^−1^]
C‐2	93.57	5.71	0.72	21.962
C‐4	94.68	4.04	1.28	81.919
C‐8	92.89	5.81	1.30	95.580
C‐10 (P‐CNSs)	90.92	7.69	1.39	549.844

To further understand the chemical process from CDs to P‐CNSs, a possible conversion mechanism are proposed. It has been demonstrated that Na‐containing compound is an effective catalyst to promote the reaction potential and weaken the C−O bond to improve the reaction rate and accelerate carbon conversion.^21^ Under high temperature and sodium catalysis, the CDs comprised of rich oxygenic functional groups were rapidly decomposed to produce abundant carbon atoms, which can then grow into carbon nanosheets by self‐assemble.^17^ The gas deriving from the rapid pyrolysis of CDs can induce instantaneous internal high pressure, which can prevent the restack of nanosheets, giving rise to the formation of ultrathin sheets.^17^, ^21^ During this process, some phosphorus atoms were simultaneously introduced into carbon nanosheets by forming P—C or P—O—C.[[qv: 13b]] Since phosphorus has a larger covalent radius of 111 pm than that of C (75 pm), S (103 pm), N (71 pm), and O (63 pm),^22^ the introduction of phosphorus into carbon material is also a quite effective approach to enlarge its interlayer distance,[[qv: 11b]] which is helpful to the Na^+^ insertion. The enlarged interlayer distance of ≈0.42 nm has been confirmed by HRTEM (Figure 3c).

**Figure 7:**
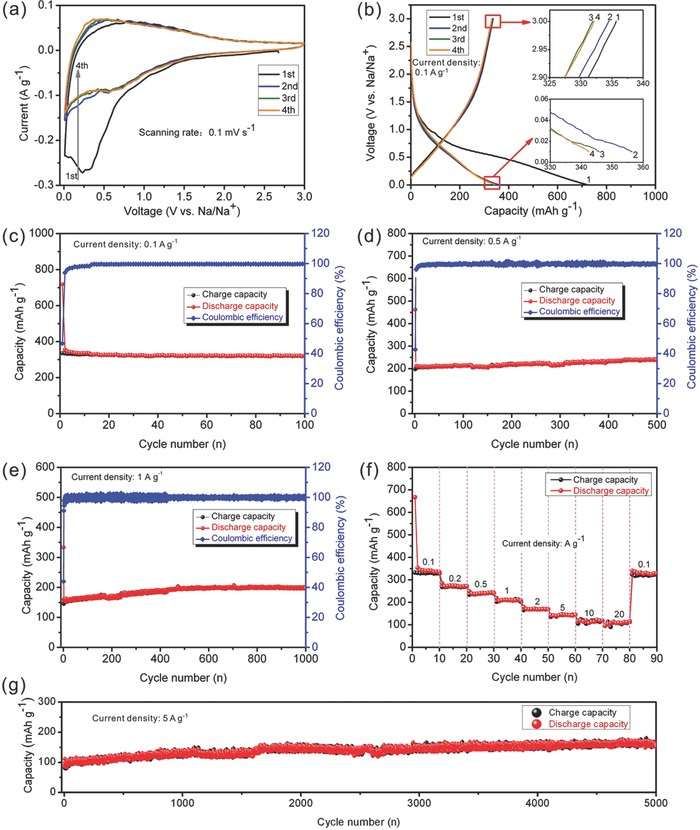
Cyclic voltammograms a), galvanostatic charge–discharge curves b), cycling performances c,d,e,g), and rate capability f) of P‐CNSs.

The P‐CNSs are utilized as electrode for half‐cells to investigate its electrochemical Na storage behaviors. **Figure** **7**a present cyclic voltammograms (CVs) of the first four cycles. The broad cathodic peak from 0.1 to 1.0 V, appearing in the first cycle, should be related to the formation of the solid electrolyte interface (SEI) film and the reaction between the Na^+^ and surface functional groups.[[qv: 7b,11a,23]] The bumps at ≈0.6 V during the cathodic process from second to fourth cycle should be ascribed to the reversible sodiation process in the surface functional groups.^17^ The cathodic peaks situated around 0.01 V is stemming from Na^+^ insertion into carbonaceous materials.^4^, ^7^, ^17^ Broad anodic peaks from 0.1 to 0.6 V in all the four cycles tells desodiation process taken place in a wide potential range. The galvanostatic charge–discharge curves at a current density of 0.1 A g^−1^ are displayed in Figure 7b. The initial discharge and charge capacities are 718.4 and 335.7 mAh g^−1^, respectively, which corresponds to a Coulombic efficiency of 46.7%. The irreversible capacity is mainly due to the formation of SEI film, the irreversible reaction between Na and surface functional groups and other side reactions.^17^ Nevertheless, the good news is that the Coulombic efficiency can rapidly approach 100% after several charge–discharge cycles. From the second cycle, the charge–discharge curves almost coincide with each other, implying the good cycling stability.

The long‐term cycling performance is a key indicator for batteries, a series of cycling tests are performed to evaluate the cyclability of P‐CNSs. When cycled at a low current density of 0.1 A g^−1^, the P‐CNSs electrode exhibits a high capacity 321.2 mAh g^−1^ after 100 cycles (Figure 7c). At a current density of 0.5 A g^−1^ (Figure 7d), the P‐CNSs deliver initial discharge and charge capacities of 462.3 and 197.2 mAh g^−1^, corresponding to a Coulombic efficiency of 42.65%. The capacity increases gradually upon cycling, originating from the activating process the porous marterials,^24^ a charge capacity can reach 237.5 mAh g^−1^ after 500 cycles. At 1 A g^−1^ (Figure 7e), the initial discharge and charge capacities are 333.3 and 146.4 mAh g^−1^, respectively, and the Coulombic efficiency is 43.92%. Similar to cycling at 0.5 A g^−1^, the capacity is rising with cycling, and the charge capacity can increase to 196.9 mAh g^−1^ after 1000 cycles. At 2.5 and 5 A g^−1^ (Figure S11, Supporting Information), the capacity is also increasing with cycling. At 2.5 A g^−1^, the charge capacity increases from 103.3 (1st cycle) to 159.9 mAh g^−1^ (2500th cycle). And at 5 A g^−1^, the charge capacity increases from 43.3 (1st cycle) to 108.8 mAh g^−1^ (5000th cycle). Nevertheless, after precycling at low current density of 0.1 A g^−1^ for five cycles, increasing the current density to 5 A g^−1^, a high capacity of 149 mAh g^−1^ can be achieved after 5000 cycles (Figure 7g), benefiting from the activated process at low current density in the first several cycles.^17^, ^24^ Figure 7f shows the rate performance of P‐CNSs electrode, the average specific capacities are 328, 269, 235, 208, 169, 143, 117, and 108 mAh g^−1^ at current densities of 0.1, 0.2, 0.5, 1, 2.5, 5, 10, and 20 A g^−1^, respectively. A high capacity of 320 mAh g^−1^ can be restored when the current density recovers to 0.1 A g^−1^, indicating a superb rate capability. As far as we know, rare carbon‐based materials have comparably accomplished durable high capabilities at such a high current density. The comparison between the P‐CNSs and the reported carbon anode for SIBs is displayed in Table S1 (Supporting Information), indicating the impressive sodium storage properties of P‐CNSs.

**Figure 8:**
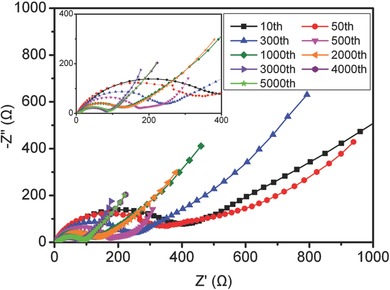
EIS of P‐CNSs at different charge–discharge cycles.

The electrochemical impedance spectra (EIS) at different cycles are employed to further explore the electrochemical sodium storage behaviors of P‐CNSs. **Figure** **8** displays the Nyquist plots, all of them are comprised of semicircles in the medium‐high frequency region and sloping straight lines in the low‐frequency range, which are ascribed to the charge‐transfer resistance and solid‐state diffusion of sodium in the electrode material, respectively. Obviously, with the increase of cycle numbers the diameters of the semicircles at medium‐high frequency region decrease, suggesting reduced charge‐transfer resistances. This may result from the special structure of P‐CNSs with high surface area and structural defects, causing large irreversible sodium insertion into the P‐CNSs during early cycles, which can enhance the electrical conductivity of the P‐CNSs.^25^ And in the subsequent cycles, the improved electrical conductivity may be attributed to the activated process of carbon‐based materials.^24^

**Figure 9:**
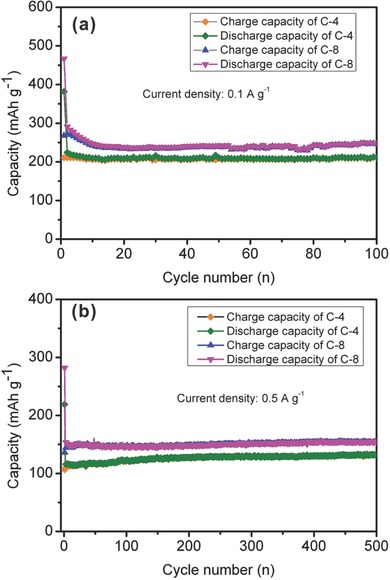
Cycling performances of samples carbonized at different mass ratios at a current density of 0.1 and 0.5 A g^−1^.

The cycling performances of the products calcined with different mass ratios were also investigated, suggesting the samples prepared at various ratios also deliver good electrochemical performances. At current density of 0.1 A g^−1^ (**Figure** **9**a), in the first cycle, the C‐8 exhibits considerable discharge and charge capacities of 268.1 and 466.6 mAh g^−1^, corresponding to an improved Coulombic efficiency of 57.46%, due to its low SSA. After 100 cycles, the charge capacity is 246.4 mAh g^−1^. Increasing the current density to 0.5 A g^−1^ (Figure 9b), the initial discharge and charge capacities are 136.3 and 281.9 mAh g^−1^, and the charge capacity increases to 154.2 mAh g^−1^ after 500 cycles. The C‐4 delivers an initial charge capacity of 210.8 mAh g^−1^ with a Coulombic efficiency of 55.19% at a current density of 0.1 A g^−1^, and 211.4 mAh g^−1^ is maintained in 100^th^ cycle (Figure 9a). At 0.5 A g^−1^ (Figure 9b), the first charge capacity of C‐4 is 106.6 mAh g^−1^, and it gradually increases to 132 mAh g^−1^ after 500 cycles.

From the above, it is proposed that the capacities rise with the increase of ratios, resulting from the decreased thickness and increased SSA, which can provide more sodium storage active sites.[[qv: 7b,9c,14b,c]] Nonetheless, the large SSA inevitably causes large irreversible reaction, giving rise to low initial Coulombic efficiency. Additionally, the P atoms covalently bonded to the carbon are electrochemically active and can be active sites for sodium storage, resulting in high reversible capacity.[[qv: 11a]] The electronic state of nanosheets was changed by the heteroatoms doping, facilitating the adsorption of electrolyte ions.[[qv: 13b]] At high current densities, the capacities of all samples increase with cycling, the increase in capacity may benefit from the activating process of the porous anode.^24^ And it is noteworthy that the Coulombic efficiency can dramatically increase to over 99% after several cycles at various current densities or all samples.

Since the morphology, structure, and composition determine the properties, the ultrathin sheets with large area, expanded graphite layer and P‐doping worked together to create the outstanding electrochemical sodium storage performances. The ultrathin nanosheets with large surface area can provide more active sites and shorten the Na^+^ diffusion length. The enlarged interlayer space is beneficial to the Na^+^ insertion and extraction. The P atoms bonded to the carbon are electrochemically active and can offer active sites for sodium sotrage. The electronic state of nanosheets was also changed by the heteroatoms doping, facilitating the adsorption of electrolyte ions.^11^, ^14^, ^16^

## 3 Conclusion

In summary, carbon dots were transformed into large‐area phosphorus‐doped carbon nanosheets with enlarged interlayer distance through thermal treatment assisted by NaH_2_PO_4_ for the first time. The detailed conversion process from carbon dots to carbon nanosheets doped with phosphorus was first explored. When applied as anode material for SIBs, the attained P‐CNSs showed remarkable electrochemical performances with high specific capacity, excellent cycling stability and ultrafast rate capability. This work proposed a novel method to fabricate phosphorus‐doped carbon nanosheets, facilitating the development of SIBs anode materials. The present work makes a significant contribution to not only the synthetic methodology of carbon materials but also the application of carbon materials as anode for SIBs.

## 4 Experimental Section

*Preparation of CDs and P‐CNSs*: 8 g sodium hydroxide (NaOH, Sinopharm Chemical Reagent Beijing Co., Ltd.) was mixed with 40 mL acetaldehyde solution (40% in H_2_O, Sinopharm Chemical Reagent Beijing Co., Ltd.) under vigorous magnetic stirring for 1 h, and then the mixture was placed at ambient air, temperature, and pressure. After 72 h, a certain amount of dilute HCl solution (1 m, Sinopharm Chemical Reagent Beijing Co., Ltd.) was added to adjust the pH to be neutral. Then the as‐prepared product was separated by centrifugation and washed with deionized water for several times, and then the final product was evaporated at 100 °C for 12 h to get CDs powder.

0.4 g of the as‐prepared CDs was fully mixed with 4 g NaH_2_PO_4_ (Sinopharm Chemical Reagent Beijing Co., Ltd.) through grind, and then the mixture was rapidly heated to 900 °C with a heating rate of 10 °C min^−1^ and calcined for 2 h. Ar atmosphere was constantly maintained through all thermal treating process. After the temperature was decreased to the room temperature, the calcined products were washed with deionized water and alcohol (Shanghai Titan Scientific Co., Ltd.) for several times and separated by vacuum suction filtration followed by drying at 100 °C for 12 h. Additionally, a series of conditional experiments were conducted to investigate the influence of calcining temperature and mass ratio of CDs to NaH_2_PO_4_ on morphology, structure, and composition. The detailed experimental parameters are displayed in **Tables** **3** and **4**.

**Table 3:** The experimental parameters at various calcining temperatures and constant mass ratio of CDs to NaH_2_PO_4_ (1:10)

Calcining temperature	Weight of CDs [g]	Weight of NaH_2_PO_4_ [g]	Marks
400 °C	0.4	4.0	C‐400
600 °C	0.4	4.0	C‐600
800 °C	0.4	4.0	C‐800
900 °C	0.4	4.0	C‐900 (P‐CNSs)

**Table 4:** The experimental parameters at various mass ratios of CDs to NaH_2_PO_4_ and constant calcining temperature (900 °C)

Ratios of CDs to NaH_2_PO_4_	Weight of CDs [g]	Weight of NaH_2_PO_4_ [g]	Marks
1:0	0.4	0	C‐0
1:2	0.4	0.8	C‐2
1:4	0.4	1.6	C‐4
1:8	0.4	3.2	C‐8
1:10	0.4	4.0	C‐10 (P‐CNSs)

*Materials Characterization*: Transmission electron microscope (JEM‐2100F), SEM (FEI Quanta 200), AFM (Dimension V, Veeco), XPS (K‐Alpha 1063), XRD (Rigaku D/max 2550 VB+ 18kW, Cu Kα radiation), Fourier transform infrared spectrophotometer (FTIR, AVTA‐TAR, 370), UV–vis spectrophotometer (UV‐1801), Raman spectroscopy (RENISHAW invia, wire 4.2), and mass spectrometer (MS) equipped with time of flight (TOF) (EI^+^) were employed to characterize the morphology, microstructure, and composition of the prepared samples. PL spectra were detected by a fluorescence spectrophotometer (F‐4500). The BET specific surface area and pore size distribution were acquired from the nitrogen adsorption/desorption technology.

*Electrochemical Test*: In order to investigate the electrochemical sodium storage behaviors, as‐prepared samples served as electrodes in half‐cells which were assembled in a glove box filled with Ar. The target materials powder were throughly mixed with conductive super P and carboxymethyl cellulose (Alfa Aesar) binder (70:15:15 in weight) in deionized water, then painting the obtained slurry on a copper foil. After the preliminary evaporation of water, the foil coated with active materials was dried at 100 °C under vacuum for 12 h. Metallic sodium (Sigma‐Aldrich) was utilized as the counter electrode, and Celgard 2400 was used as the separator. The solution of 1 m NaClO_4_ (Alfa Aesar) in PC (Sigma‐Aldrich) with 5% fluoroethylene carbonate (Alfa Aesar) additive was employed as electrolyte. Solartron Analytical was employed to collect cyclic voltammograms (0.01–3 V, vs Na/Na^+^, 0.1 mV s^−1^) and EIS (100 kHz to 0.01 Hz). Arbin battery cycler (BT2000) was used to test charge–discharge performances at suitable current densities at room temperature.

## Supporting information

As a service to our authors and readers, this journal provides supporting information supplied by the authors. Such materials are peer reviewed and may be re‐organized for online delivery, but are not copy‐edited or typeset. Technical support issues arising from supporting information (other than missing files) should be addressed to the authors.

Supplementary

## References

[1] a) D. Kundu , E. Talaie , V. Duffort , L. F. Nazar , Angew. Chem., Int. Ed. 2015, 54, 3431; 10.1002/anie.201410376 25653194

[2] a) M.‐S. Balogun , Y. Luo , W. Qiu , P. Liu , Y. Tong , Carbon 2016, 98, 162;

[3] a) M. M. Doeff , Y. Ma , S. J. Visco , L. C. De Jonghe , J. Electrochem. Soc. 1993, 140, L169;

[4] a) K. Tang , L. Fu , R. J. White , L. Yu , M.‐M. Titirici , M. Antonietti , J. Maier , Adv. Energy Mater. 2012, 2, 873;

[5] D. Li , H. Chen , G. Liu , M. Wei , L.‐X. Ding , S. Wang , H. Wang , Carbon 2015, 94, 888.

[6] a) Y. Liu , F. Fan , J. Wang , Y. Liu , H. Chen , K. L. Jungjohann , Y. Xu , Y. Zhu , D. Bigio , T. Zhu , C. Wang , Nano Lett. 2014, 14, 3445; 24823874 10.1021/nl500970a

[7] a) J. Ding , H. Wang , Z. Li , A. Kohandehghan , K. Cui , Z. Xu , B. Zahiri , X. Tan , E. M. Lotfabad , B. C. Olsen , D. Mitlin , ACS Nano 2013, 7, 11004; 24191681 10.1021/nn404640c

[8] a) Y. Yan , Y.‐X. Yin , Y.‐G. Guo , L.‐J. Wan , Adv. Energy Mater. 2014, 4, 1301584;

[9] a) S. Komaba , W. Murata , T. Ishikawa , N. Yabuuchi , T. Ozeki , T. Nakayama , A. Ogata , K. Gotoh , K. Fujiwara , Adv. Funct. Mater. 2011, 21, 3859;

[10] a) X. Wang , L. Lv , Z. Cheng , J. Gao , L. Dong , C. Hu , L. Qu , Adv. Energy Mater. 2016;

[11] a) W. Li , M. Zhou , H. Li , K. Wang , S. Cheng , K. Jiang , Energy Environ. Sci. 2015, 8, 2916;

[12] C. Bommier , T. W. Surta , M. Dolgos , X. Ji , Nano Lett. 2015, 15, 5888. 26241159 10.1021/acs.nanolett.5b01969

[13] a) J. Duan , S. Chen , M. Jaroniec , S. Z. Qiao , ACS Catal. 2015, 5, 5207;

[14] a) X. Zheng , J. Luo , W. Lv , D.‐W. Wang , Q.‐H. Yang , Adv. Mater. 2015, 27, 5388; 26207982 10.1002/adma.201501452

[15] a) S. Y. Lim , W. Shen , Z. Gao , Chem. Soc. Rev. 2015, 44, 362; 25316556 10.1039/c4cs00269e

[16] J. Deng , Q. Lu , N. Mi , H. Li , M. Liu , M. Xu , L. Tan , Q. Xie , Y. Zhang , S. Yao , Chem. Eur. J. 2014, 20, 4993. 24623706 10.1002/chem.201304869

[17] H. Hou , C. E. Banks , M. Jing , Y. Zhang , X. Ji , Adv. Mater. 2015, 27, 7861. 26506218 10.1002/adma.201503816

[18] X. Chen , Q. Jin , L. Wu , C. Tung , X. Tang , Angew. Chem., Int. Ed. 2014, 53, 12542. 10.1002/anie.201408422 25296956

[19] M. Latorre‐Sanchez , A. Primo , H. Garcia , Angew. Chem., Int. Ed. 2013, 52, 11813. 10.1002/anie.201304505 24105902

[20] a) J. Song , Z. Yu , M. L. Gordin , X. Li , H. Peng , D. Wang , ACS Nano 2015, 9, 11933; 26498828 10.1021/acsnano.5b04474

[21] H. Cui , J. Zheng , P. Yang , Y. Zhu , Z. Wang , Z. Zhu , ACS Appl. Mater. Interfaces 2015, 7, 11230. 25961810 10.1021/acsami.5b01201

[22] P. Pyykko , M. Atsumi , Chem. Eur. J. 2009, 15, 186. 19058281 10.1002/chem.200800987

[23] L. Fu , K. Tang , K. Song , P. A. van Aken , Y. Yu , J. Maier , Nanoscale 2014, 6, 1384. 24306060 10.1039/c3nr05374a

[24] L. Qie , W.‐M. Chen , Z.‐H. Wang , Q.‐G. Shao , X. Li , L.‐X. Yuan , X.‐L. Hu , W.‐X. Zhang , Y.‐H. Huang , Adv. Mater. 2012, 24, 2047. 22422374 10.1002/adma.201104634

[25] R. Song , H. Song , J. Zhou , X. Chen , B. Wu , H. Y. Yang , J. Mater. Chem. 2012, 22, 12369.

