# Supplementary material for: Large‐Area Carbon Nanosheets Doped with Phosphorus: A High‐Performance Anode Material for Sodium‐Ion Batteries
Source: Adv Sci (Weinh). 2016 Sep 12;4(1):1600243. doi: 10.1002/advs.201600243 (PMC5238737; doi:10.1002/advs.201600243)
Supplement: Supplementary file 1 — Supplementary [file ADVS-4-0-s001.pdf]

Copyright WILEY-VCH Verlag GmbH & Co. KGaA, 69469 Weinheim, Germany, 2016.

ADVANCED SCIENCE
Open Access

Supporting Information

for *Adv. Sci.,* DOI: 10.1002/advs.201600243

Large-Area Carbon Nanosheets Doped with Phosphorus: A High-Performance Anode Material for Sodium-Ion Batteries

*Hongshuai Hou, Lidong Shao, Yan Zhang, Guoqiang Zou, Jun Chen, and Xiaobo Ji**

Copyright WILEY-VCH Verlag GmbH & Co. KGaA, 69469 Weinheim, Germany, 2013.

Supporting Information

Large-area Carbon Nanosheets Doped with Phosphorus：a High-performance Anode Material for Sodium-ion Batteries

*Hongshuai Hou, Lidong Shao, Yan Zhang, Guoqiang Zou, Jun Chen, and Xiaobo Ji**

H. Hou, Y. Zhang, G. Zou, J. Chen, and Prof. X. Ji

College of Chemistry and Chemical Engineering

Central South University

Changsha,410083, China

E-mail: xji@csu.edu.cn

Prof. L. Shao

Shanghai Key Laboratory of Materials Protection and Advanced Materials in Electric Power,

Shanghai University of Electric Power, Shanghai 200090, China

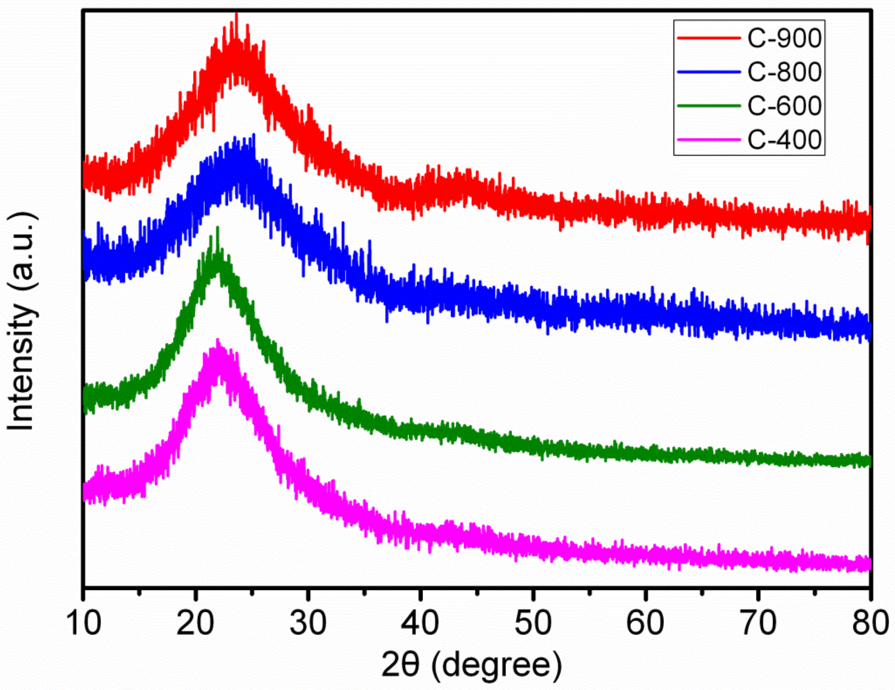

Figure S1. The XRD patterns of samples carbonized at different temperatures.

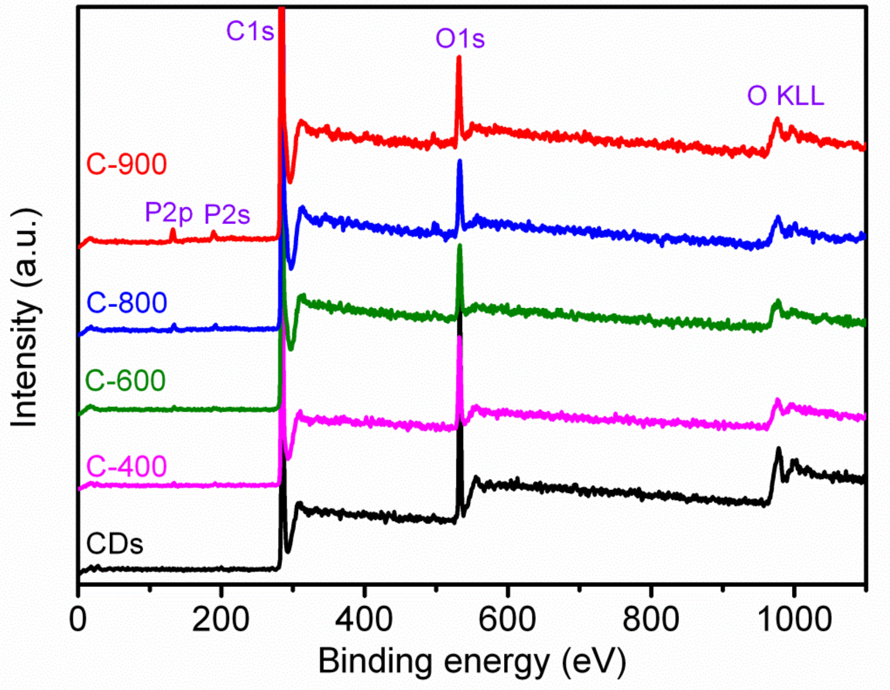

Figure S2. The survey XPS spectra of samples carbonized at different temperatures.

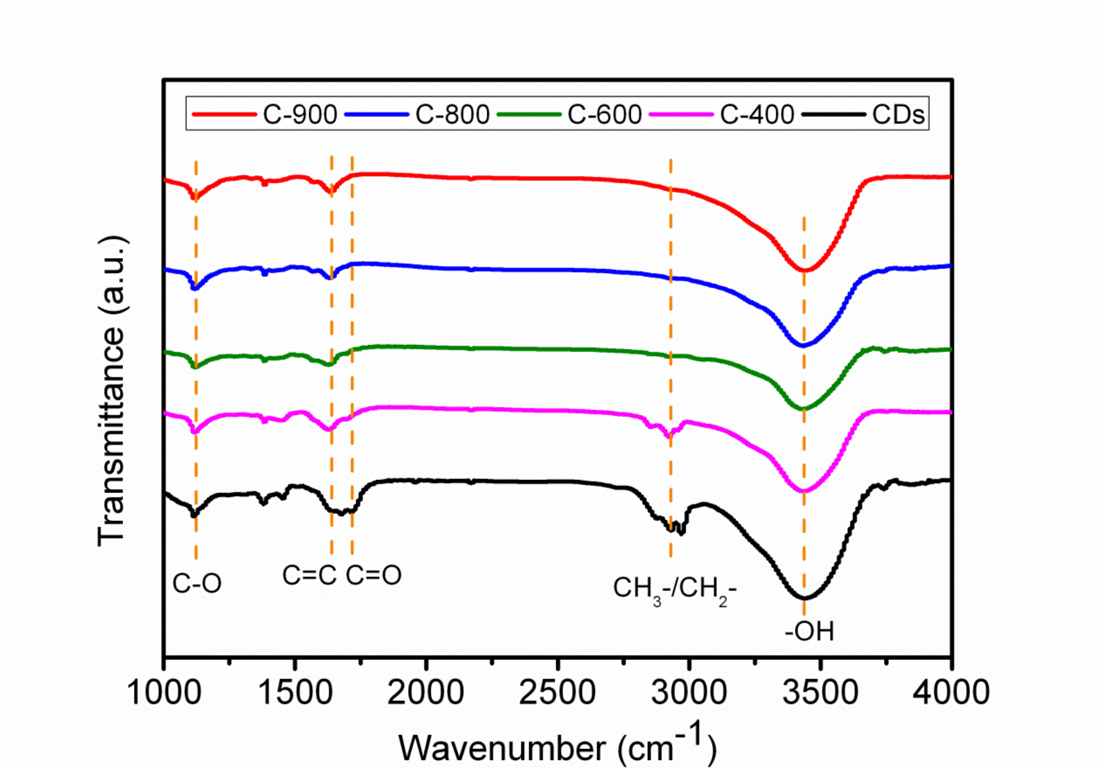

Figure S3. The FTIR spectra of samples carbonized at different temperatures.

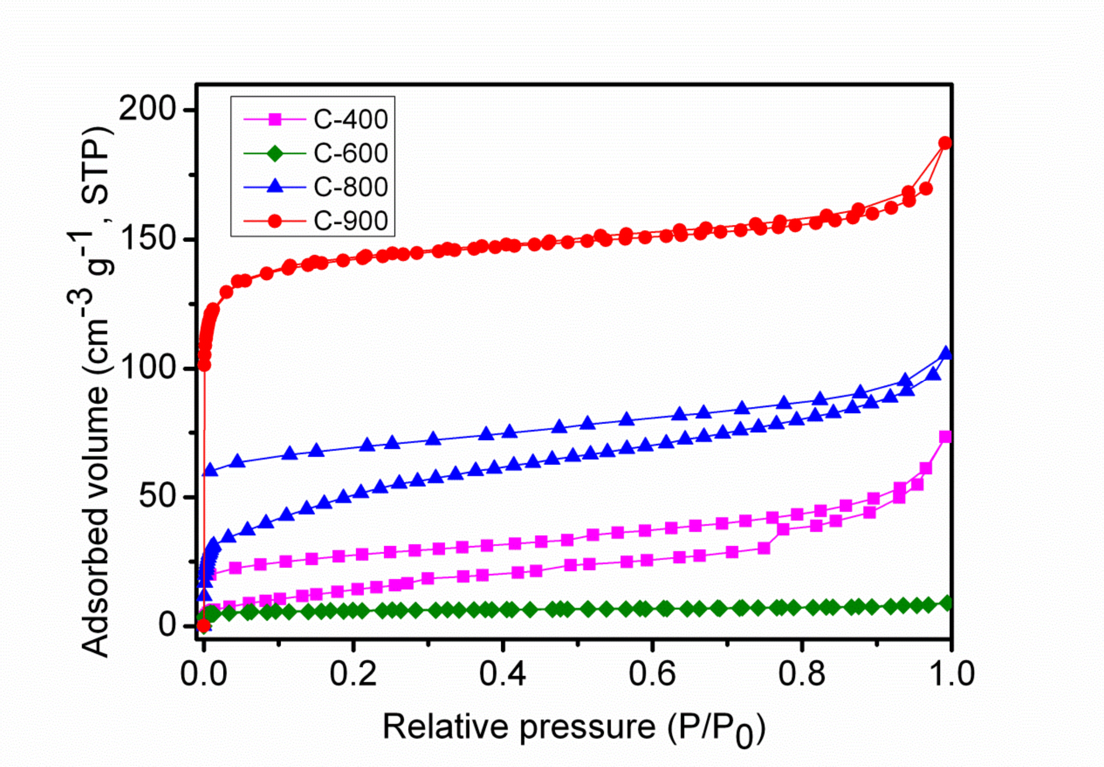

Figure S4. Nitrogen adsorption-desorption isotherms of samples carbonized at different temperatures.

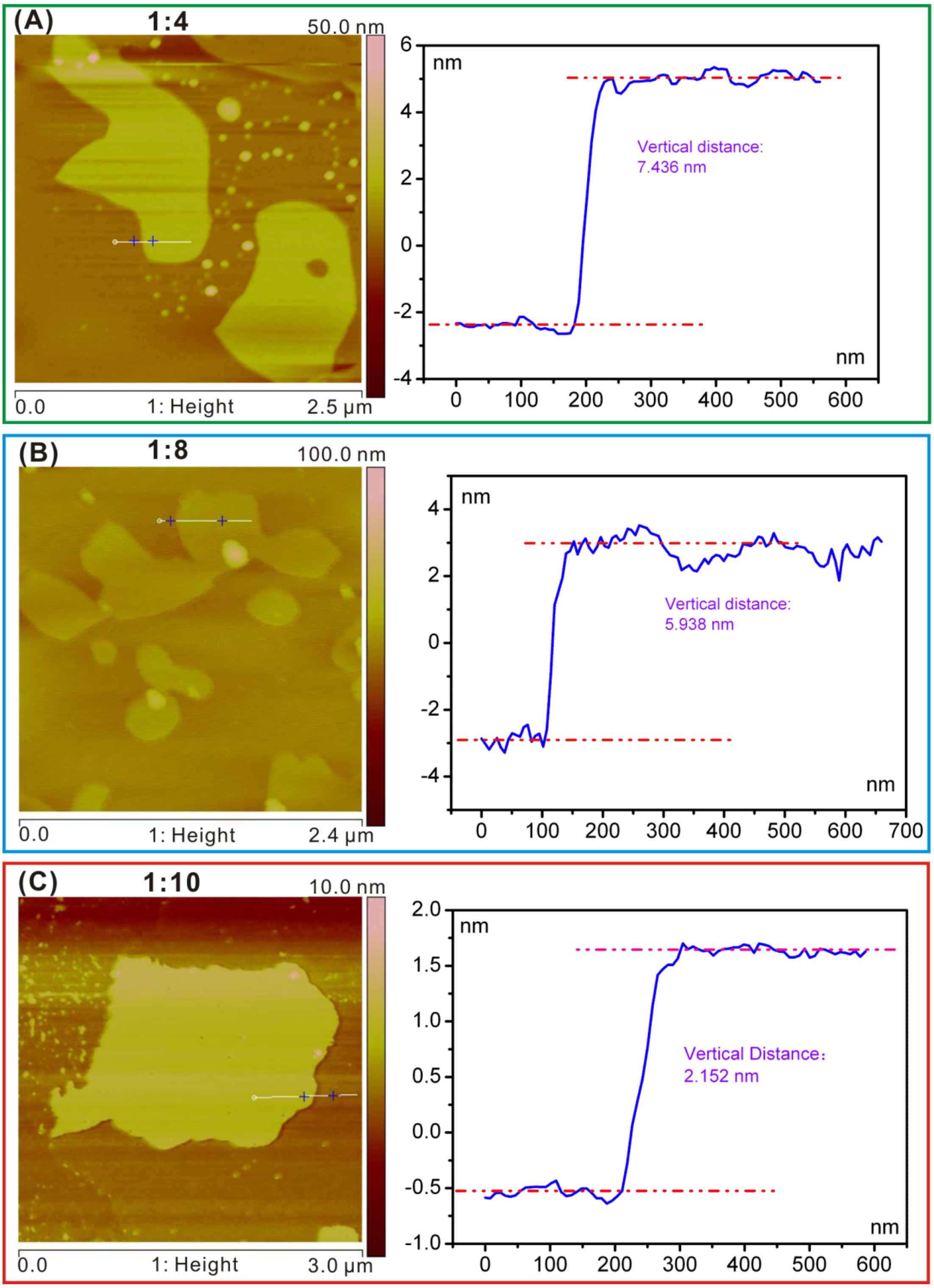

Figure S5. AFM results of samples carbonized at different mass ratios of CDs to NaH_2PO_4.

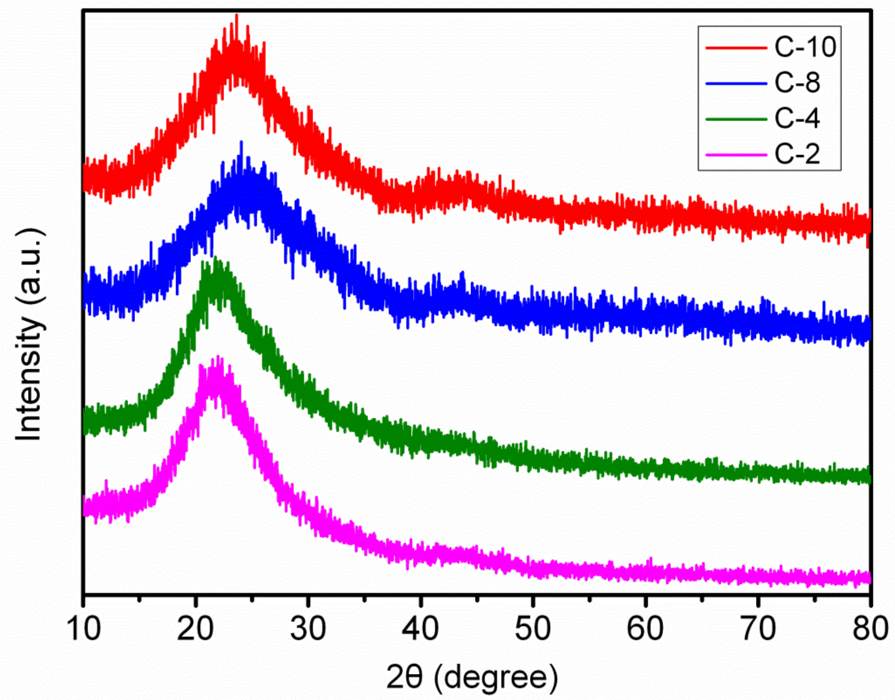

Figure S6. The XRD patterns of samples carbonized at different mass ratios of CDs to NaH_2PO_4.

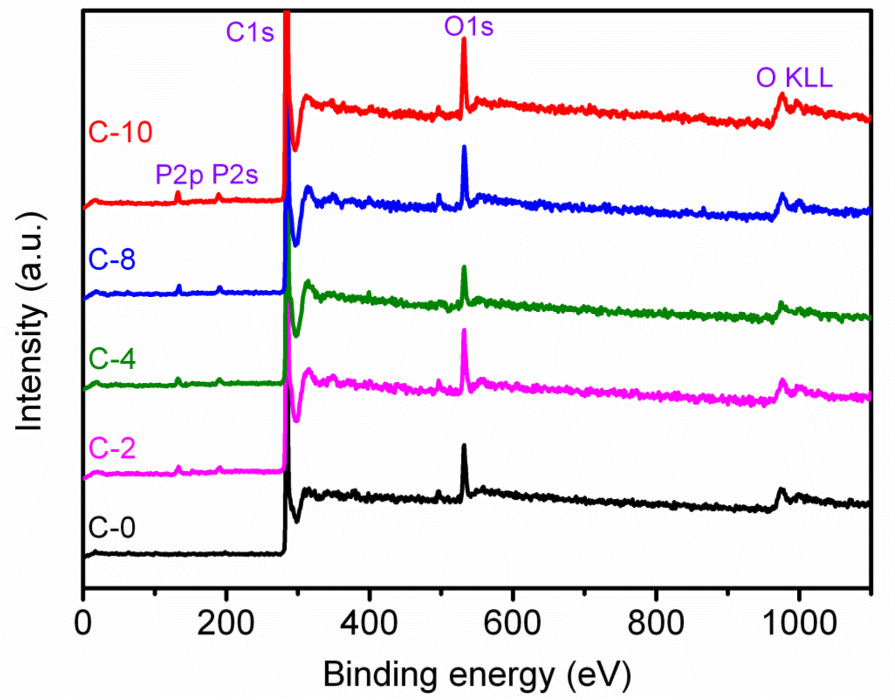

Figure S7. The survey XPS spectra of samples carbonized at different mass ratios of CDs to NaH_2PO_4.

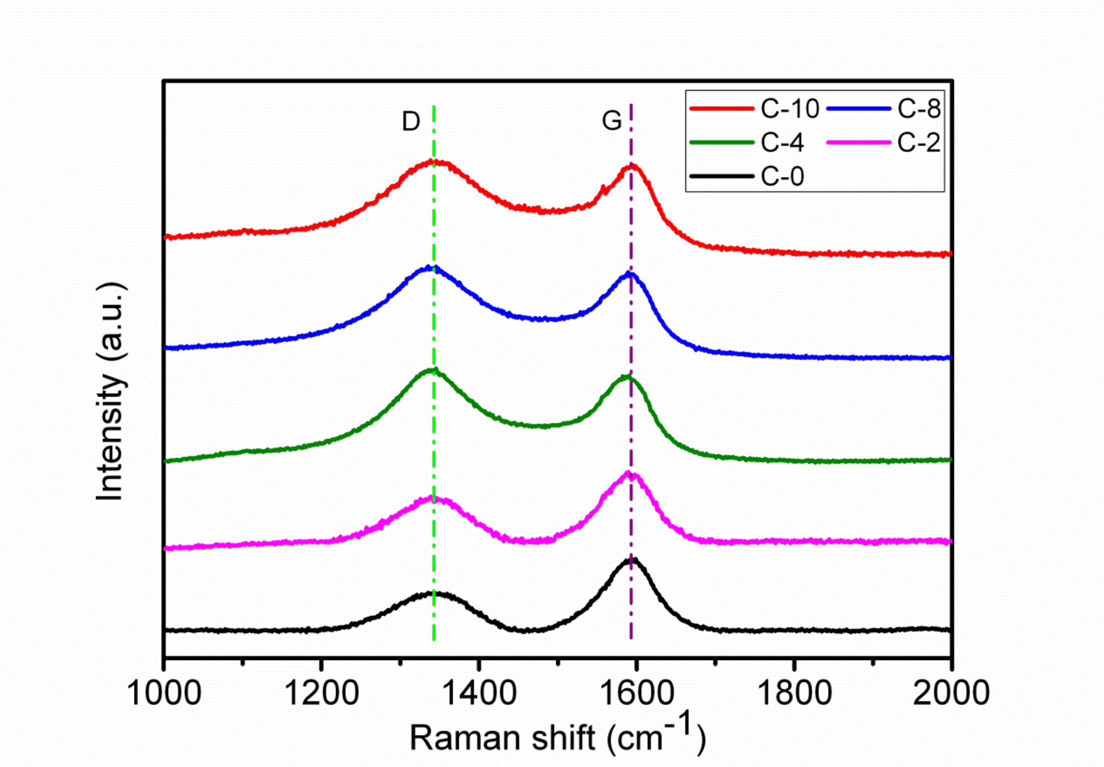

Figure S8. The Raman spectra of samples carbonized at different mass ratios of CDs to NaH_2PO_4.

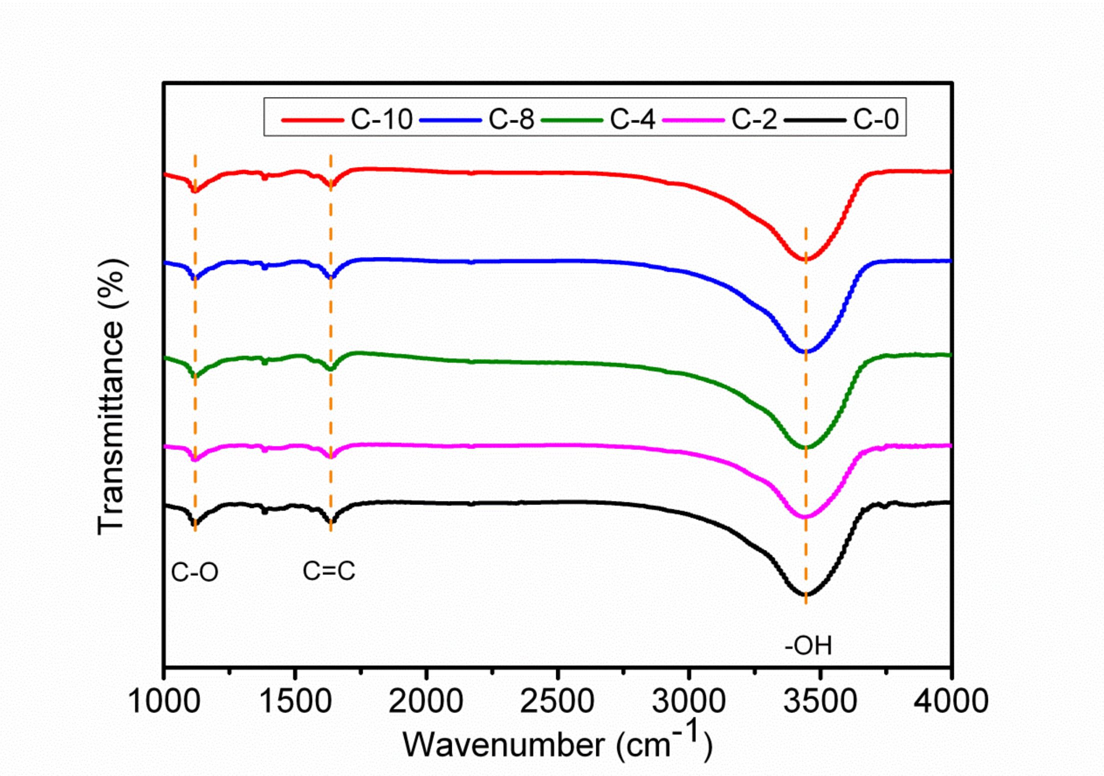

Figure S9. The FTIR spectra of samples carbonized at different mass ratios of CDs to NaH_2PO_4.

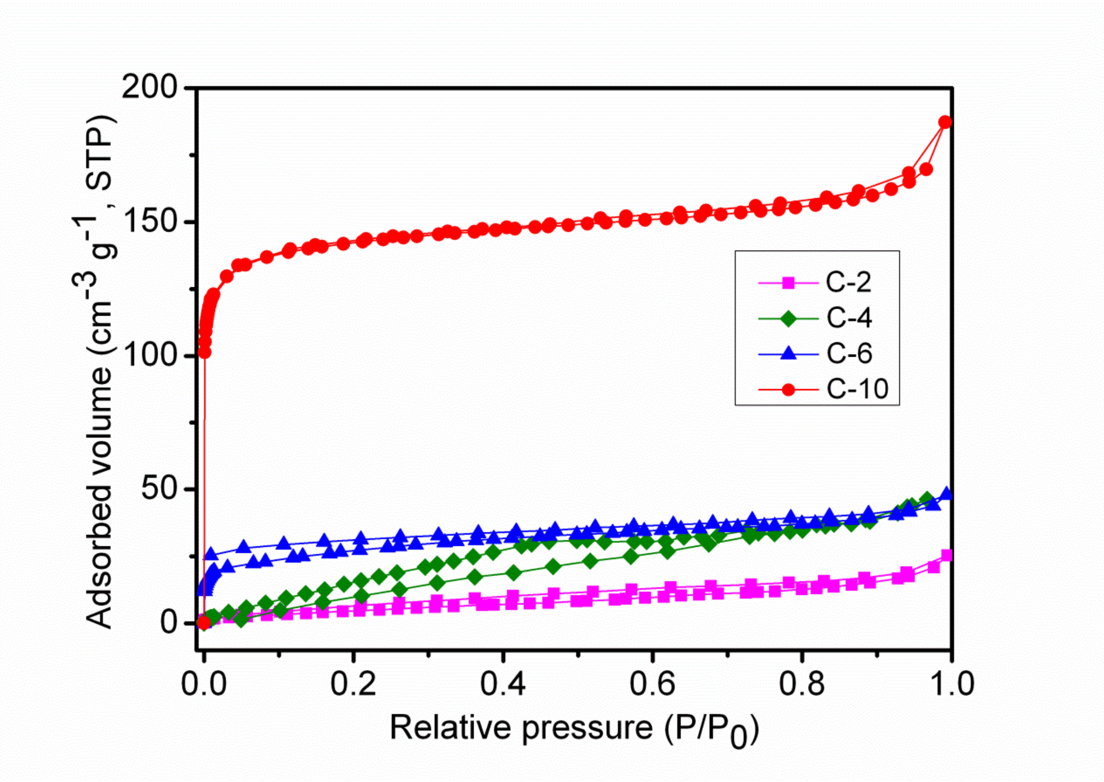

Figure S10. Nitrogen adsorption-desorption isotherms of samples carbonized at different mass ratios of CDs to NaH_2PO_4.

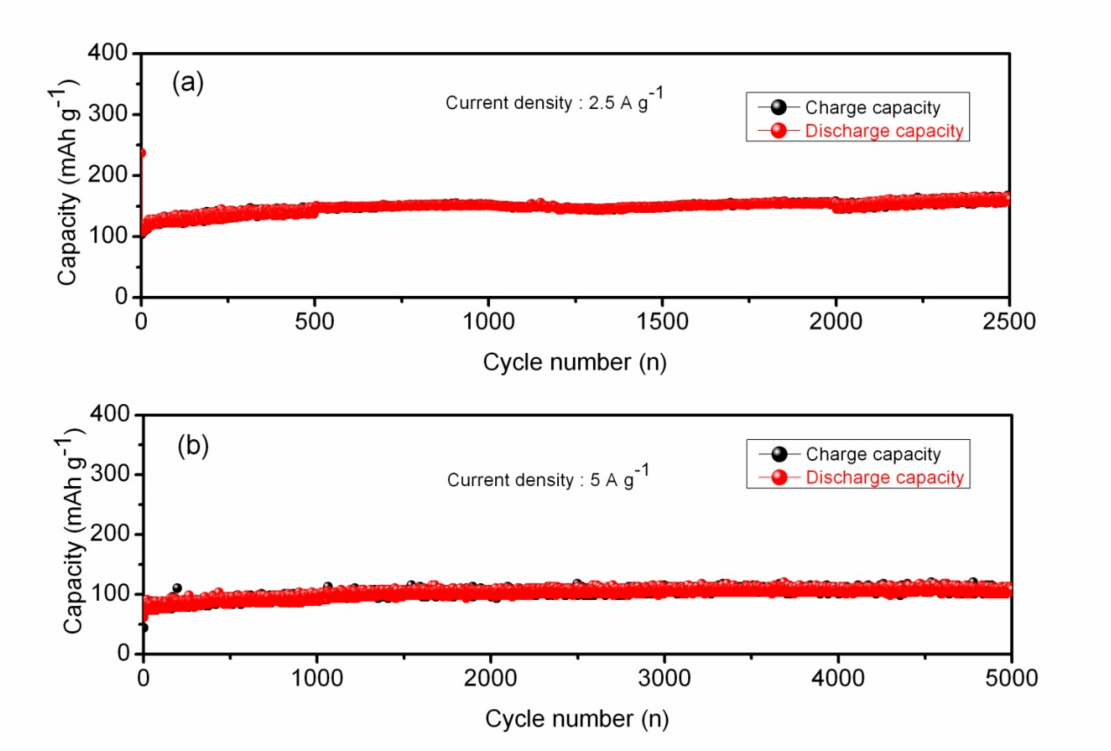

Figure S11. Cycling performances of P-CNSs at current densities of 2.5 and 5 A g^{-1}.

Table S1. Cayclability and rate capability comparison of P-CNSs *vs.* reported SIBs carbon anode materials.

Materials	Cyclability (mAh g^{-1})	Rate capability (mAh g^{-1})	References
P-CNSs	**321.2 at 0.1 A g^{-1} after 100 cycles;** 237.5 at 0.5 A g^{-1} after 500 cycles; 159.9 at 2.5 A g^{-1} after 2500 cycles; **108.8 at 5 A g^{-1} after 5000 cycles**	269 at 0.2 A g^{-1} ; 235 at 0.5 A g^{-1} ; 208 at 1 A g^{-1} ; 169 at 2A g^{-1} ; 143 at 5 A g^{-1} ; 117 at 10 A g^{-1} ; **108 at 20 A g^{-1}**	This work
hard carbon	225 at 0.025 A g^{-1} after 100 cycles	No reported	S1
templated carbon	120 at 0.074 A g^{-1} after 40 cycles	~ 140 at 0.074 A g^{-1}; ~ 120 at 0.74 A g^{-1}; ~ 100 at 1.85 A g^{-1}	S2
hollow carbon nanospheres	160 at 0.1 A g^{-1} after 100 cycles	168 at 0.2 A g^{-1}; 142 at 0.5 A g^{-1}; 120 at 1 A g^{-1}; 100 at 2 A g^{-1}; 75 at 5 A g^{-1}	S3
hollow carbon nanowires	~ 220 at 0.05 A g^{-1} after 200 cycles	210 at 0.25 A g^{-1}; 149 at 0.5 A g^{-1}	S4
carbonized peat moss	255 at 0.1 A g^{-1} after 210 cycles	250 at 0.2 mA g^{-1}; 203 at 0.5 A g^{-1}; 150 at 1 A g^{-1}; 106 at 2 A g^{-1}; 66 at 5 A g^{-1}	S5
nanocellular carbon foams	137 at 0.1 A g^{-1} after 300 cycles	140 at 0.2 A g^{-1}; 120 at 0.5 A g^{-1}; 100 at 1 A g^{-1}; 50 at 5 A g^{-1}	S6
carbon nanosheets	155 at 0.05 A g^{-1} after 200 cycles	~ 190 at 0.2 A g^{-1}; ~ 125 at 0.5 A g^{-1}; ~ 80 at 1 A g^{-1}; 50 at 2 A g^{-1}; 45 at 5 A g^{-1}	S7
carbon nanofibers	134.2 at 0.2 A g^{-1} after 200 cyels	150 at 0.2 A g^{-1}; 139 at 0.5 A g^{-1}; 132 at 1 A g^{-1}; 121 at 2 A g^{-1}; 100 at 5 A g^{-1}	S8
carbon nanofibers	243 at 0.05 A g^{-1} after 100 cycles	210 at 0.2 A g^{-1}; 175 at 0.5A g^{-1}; 153 at 1 A g^{-1}; 134 at 2 A g^{-1}; 101 at 5 A g^{-1}	S9
carbon nanofibers	~ 260 at0.05 A g^{-1} after 280 cycles	No reported	S10
banana peel pseudographite	298 at 0.1 A g^{-1} after 300 cycles	290 at 0.2 A g^{-1}; 238 at 0.5 A g^{-1}; 155 at 1 A g^{-1}; 100 at 2 A g^{-1}; 70 at 5 A g^{-1}	S11
porous carbon/graphene composite	250 at 1 A g^{-1} after 1000 cycles	No reported	S12
natural graphite	127 at 0.1 A g^{-1} after 300 cycles; ~ 100 at 0.5 A g^{-1} after 2500 cycles	~ 145 at 0.2 A g^{-1}; ~ 137 at 0.5 A g^{-1}; ~ 128 at 1 A g^{-1}; ~ 112 at 3 A g^{-1}; ~ 103 at 5 A g^{-1};	S13

		~ 78 at 10 A g^{-1}	
amorphous carbon/graphene composite	142 at 0.5 A g^{-1} after 2500 cycles	230 at 0.1 A g^{-1}; ~ 180 at 0.5 A g^{-1}; ~ 170 at 1 A g^{-1}; ~ 150 at 2 A g^{-1}; ~ 130 at 5 A g^{-1}; 120 at 10 A g^{-1}	S14
3D porous carbon frameworks	303.2 at 0.1 A g^{-1} after 100 cycles; 256.5 at 0.5 A g^{-1} after 500 cycles; 147.3 at 2.5 A g^{-1} after 2500 cycles; 98.3 at 5 A g^{-1} after 5000cycles	290 at 0.2 A g^{-1} ; 253 at 0.5 A g^{-1} ; 200 at 1 A g^{-1} ; 166 at 2A g^{-1} ; 130 at 5 A g^{-1} ; 104 at 10 A g^{-1} ; 90 at 20 A g^{-1}	S15
hierarchical N/S-codoped carbon	150 at 0.5 A g^{-1} after 3400 cycles	280 at 0.03 A g^{-1} ; 210 at 0.1 A g^{-1} ; 180 at 0.2 A g^{-1} ; 155 at 0.5 A g^{-1} ; 143 at 1 A g^{-1} ; 132 at 2.5 A g^{-1} ; 131 at 5 A g^{-1}	S16
hard carbon microtubes	305 at 0.03 A g^{-1} after 100 cycles	275 at 0.15 A g^{-1} ; 180 at 0.3 A g^{-1}	S17

S1) S. Komaba, W. Murata, T. Ishikawa, N. Yabuuchi, T. Ozeki, T. Nakayama, A. Ogata, K. Gotoh, K. Fujiwara, *Adv. Funct. Mater.* **2011**, *21*, 3859.

S2) S. Wenzel, T. Hara, J. Janek, P. Adelhelm, *Energy Environ. Sci.* **2011**, *4*, 3342.

S3) K. Tang, L. Fu, R. J. White, L. Yu, M. M. Titirici, M. Antonietti, J. Maier, *Adv. Energy Mater.* **2012**, *2*, 873.

S4) Y. Cao, L. Xiao, M. L. Sushko, W. Wang, B. Schwenzer, J. Xiao, Z. Nie, L. V. Saraf, Z. Yang, J. Liu, *Nano Lett.* **2012**, *12*, 3783.

S5) J. Ding, H. Wang, Z. Li, A. Kohandehghan, K. Cui, Z. Xu, B. Zahiri, X. Tan, E. M. Lotfabad, B. C. Olsen, D. Mitlin, *ACS Nano* **2013**, *7*, 11004.

S6) Y. Shao, J. Xiao, W. Wang, M. Engelhard, X. Chen, Z. Nie, M. Gu, L. V. Saraf, G. Exarhos, J. G. Zhang, J. Liu, *Nano Lett.* **2013**, *13*, 3909.

S7) H. Wang, Z. Wu, F. Meng, D. Ma, X. Huang, L. Wang, X. Zhang, *Chemsuschem* **2013**, *6*, 56.

S8) Z. Wang, L. Qie, L. Yuan, W. Zhang, X. Hu, Y. Huang, *Carbon* **2013**, *55*, 328.

S9) L. Fu, K. Tang, K. Song, P. A. van Aken, Y. Yu, J. Maier, *Nanoscale* **2014**, *6*, 1384.

S10) Y. Liu, F. Fan, J. Wang, Y. Liu, H. Chen, K. L. Jungjohann, Y. Xu, Y. Zhu, D. Bigio, T. Zhu, C. Wang, *Nano Lett.* **2014**, *14*, 3445.

S11) E. M. Lotfabad, J. Ding, K. Cui, A. Kohandehghan, W. P. Kalisvaart, M. Hazelton, D. Mitlin, *ACS Nano* **2014**, *8*, 7115.

S12) Y. Yan, Y. X. Yin, Y. G. Guo, L. J. Wan, *Adv. Energy Mater.* **2014**, *4*, 1301584.

S13) H. Kim, J. Hong, Y. U. Park, J. Kim, I. Hwang, K. Kang, *Adv. Funct. Mater.* **2015**, *25*, 534.

S14) S. Li, J. Qiu, C. Lai, M. Ling, H. Zhao, S. Zhang, *Nano Energy* **2015**, *12*, 224.

S15) H. Hou, C. E. Banks, M. Jing, Y. Zhang, X. Ji, *Adv. Mater.* **2015**, 27, 7861.

S16) D. Xu, C. Chen, J. Xie, B. Zhang, L. Miao, J. Cai, Y. Huang, L. Zhang, Adv. Energy Mater. **2016**, 6, 1501929.

S17) Y. Li, Y. S. Hu, M. M. Titirici, L. Chen, X. Huang, Adv. Energy Mater. **2016**, DOI: 10.1002/aenm.201600659.
